# A Laboratory Machine Verifying the Operation of a Hydraulic Rope Equalizer with Tensometric Sensors

**DOI:** 10.3390/s24082588

**Published:** 2024-04-18

**Authors:** Leopold Hrabovský, Jiří Fries, Štěpán Pravda

**Affiliations:** Department of Machine and Industrial Design, Faculty of Mechanical Engineering, VSB—Technical University of Ostrava, 17. listopadu 2172/15, 708 00 Ostrava, Czech Republic; jiri.fries@vsb.cz (J.F.); stepan.pravda@vsb.cz (Š.P.)

**Keywords:** tensometric sensor, rope equalizer, mining machine, traction elevator, breaking force, stranded rope

## Abstract

In mining machines with friction discs, but also in multi-rope traction elevators, it is necessary to distribute the applied tensile load, generated by the weight of the cage and counterweight, evenly in all cross-sections of the load-bearing ropes. Hydraulic devices used for this purpose can operate on the principle of Pascal’s law. This article presents a structural design, a 3D model and an implemented solution of a laboratory device capable of simulating a practical method of evenly distributing the total weight of the load into partial tensile forces of the same size acting on a selected number of load-bearing ropes. The laboratory equipment uses two pairs of three steel cables of finite length for the simulations. During the experimental measurements, tensile forces derived from the tractive force of the piston rods, pushed into the bodies of the hydraulic cylinders by the pressure of the hydraulic oil supplied through the pipeline under the pistons of the hydraulic cylinders, were detected. The resulting amount of hydraulic oil pressure in the hydraulic circuit influenced by different values of the hydraulic oil pressures in the hydraulic cylinders and by the pressure in the supply pipe was experimentally studied on the laboratory equipment. Simulations were also carried out in order to detect the hydraulic oil pressure in the hydraulic circuit caused by the change in the different magnitudes of the tensile forces in the ropes. From the experiments carried out, it follows that with the appropriate choice of hydraulic elements and the design of the hydraulic circuit, the weight of the load, acting as the total pulling force in the ropes, can be evenly distributed (with a deviation of up to 5%) to all cross-sections of the load-bearing ropes. If the exact values of the hydraulic oil volumes under the pistons of all hydraulic cylinders are not known, it is not possible to calculate the pressure values in the hydraulic circuit when the valves of the hydraulic pipes are gradually opened.

## 1. Introduction

In [1], it is stated that cages, balancing or leveling weights must be suspended on steel ropes. The ropes must meet two requirements: (a) the nominal diameter must be at least 8 mm and (b) the nominal tensile strength of the wires and their parameters must be as specified in EN 123855:2002 [2].

With the entry of the Czech Republic into the EU in 2004, “new” European legislation in the field of testing and placing products on the European market began to apply in the Czech Republic as well. The task of the “New Approach” to European technical legislation is to remove barriers to trade and prevent dangerous products from entering the European market [3].

The European standardization bodies (CEN, CENELEC, ETSI) issue so-called harmonized standards in support of the directives. Compliance with these standards is voluntary. The manufacturer or importer may produce/supply products on the market according to standards other than harmonized standards, but these must be in accordance with the relevant directive. Meeting the requirements of a harmonized standard automatically means that the product is safe and is considered to comply with the directive, which is binding.

The area of steel ropes is not covered by a single document but rather falls under several directives depending on the use of the rope: (a) elevators, (b) machinery, (c) passenger cable cars and lifts or (d) mining traction machines. For each of the aforementioned areas, different requirements (combinations of modules) for conformity assessment and product certification apply in accordance with the relevant directives and the Act on Technical Requirements for Products (No. 22/1997 Coll.).

Conformity assessment procedures according to Act 22/1997 Coll. [4] represent the minimum requirements according to which the conformity assessment and certification of the product should be carried out; in the case of an elevator, it is a combination of modules B + C.

Module B: The assessment of the conformity of a product type sample (prototype) by an authorized person and issuance of a certificate by an authorized person (product type certification): (a) the manufacturer or importer provides the authorized person with technical documentation and a representative sample of the product type; (b) the authorized person performs tests to see if the product type sample meets the requirements of technical regulations and issues an EC type approval certificate (product type certificate).

Module C: The assessment of the conformity of the product with the certified product type: the manufacturer, importer and authorized person proceed according to module B and additionally, (a) the manufacturer or importer issues a declaration of conformity, (b) the importer takes all measures to ensure the conformity of the product properties with the certified type and (c) the authorized person performs the testing of product properties at random intervals for the purpose of verifying conformity, as stipulated by the relevant technical regulation.

From the above, it follows that steel ropes require different levels of conformity assessment and product certification depending on the method of use. In terms of the “new” European legislation, the minimum is to submit technical documentation and a representative sample of the product to an authorized person who will perform tests and issue an EC type approval certificate. The manufacturer or importer then issues a declaration of conformity (certificate) upon the delivery of the product. This procedure applies to ropes in elevators.

Chapter “1.3 Suspension and support devices” of “Appendix No. 1 to Government Regulation No. 122/2016 Coll.” [4] states the following: Suspension or support devices of the elevator cage, its fastening and connecting parts must be selected and designed so as to ensure the necessary level of safety and minimize the risk of the cage falling, taking into account the conditions of use, the materials used and the manufacturing conditions. Where ropes or chains are used to suspend the cage, a minimum of two separate ropes or chains, each with a separate fastening, must be used. These ropes and chains shall not have any joints or entanglements unless necessary for fastening or to form loops.

Given that compliance with harmonized standards is voluntary, it is possible to use ropes with diameters smaller than d_r_ = 8 mm in traction elevators intended for the transport of persons, or persons and cargo, according to [5], if compliance is assessed according to [4,5].

According to [1], the load capacity of traction elevators [6,7,8], which are adapted to transport people, is assigned the largest usable area of the cage, in order to prevent the overloading of the cage with the number of transported persons. For high load capacities (over 2500 kg) of traction elevators, 0.16 m^2^ of area is added (to an area of 5 m^2^) for every 100 kg increase in weight.

According to Relation (1), it is possible to determine the required number of load-bearing ropes, n_r_ [−], if Q [kg]—elevator capacity, K [kg]—cage weight, i_t_—rope transmission [−], k_r_ [−] rope safety and F_min_ [N]—minimum breaking force at rope tensile strength S [N·mm^−2^]—are known. The large number of load-bearing ropes results from the necessity of choosing smaller rope diameters, as these diameters determine the minimum diameters of the friction discs (40 times) and rope pulleys.

(1) $$n_{r}=\frac{\left(Q+K\right)\cdot g}{i_{t}\cdot k_{r}\cdot F_{\min}}\;[-],$$

**Figure 1:**
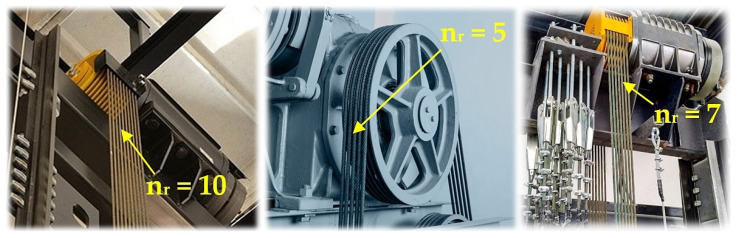
Load-bearing ropes of traction elevators guided by the grooves of the friction disc.

According to Relation (1), it can be observed that (for the chosen design of the nominal diameter wire rope d_r_ [m], which is assigned according to [2] the minimum breaking force F_min_ [N] of the rope at the rope grade S [N · m^−2^]) the number of load-bearing ropes of the elevator n_r_ [−] is directly proportional to the lifting capacity Q [kg]. With high lifting capacities Q [kg], it is necessary to hang the cage on a large number of ropes [9,10] in traction elevators; see Figure 1.

However, an increasing number of the cross-sections of carrier ropes leads to a possible situation, when the loading exerted by the weight of the cage and the weight of the counterweight is not evenly distributed into the individual cross-sections of the carrier ropes. This different loading of the individual carrier ropes leads to an uneven wear of the friction disc grooves and different wear of the individual carrier ropes.

The equalization of tensile forces in the cross-sections of the steel ropes of traction elevators is based on a mechanical [11,12,13,14] or hydraulic [15,16] principle.

Foreign scholars have conducted a lot of work on monitoring the wire rope tension and load. In foreign countries, the wire rope tension and load measurement method measured by the sensor or other instruments have been developed in many countries [17,18,19].

In 2012, Jin and Zhang [20] designed a tension sensor for the hoist wire rope using the strain gauge as the measurement principle and the spoke as the flexible structure and adopted configuration software as detection system software to realize the online monitoring of the tension.

G. Lei et al. in the article [21] state that due to the longitudinal and lateral coupling vibration of wire ropes during the operation of the hoist, there are high-frequency components in measured tension signals of wire ropes, which cannot effectively characterize the actual lifting load. To overcome this problem, a particle damping sensor with a vibration dissipation function is designed.

L. Hrabovský in [22] presents the construction design, 3D model and produced device of one of four produced prototypes, which were constructed in the “Research and Testing Laboratory” and allow for the setting of differing values of tension forces in the system of the ropes of a traction elevator to values of the same size.

In the study [23], M. Yao et al. designed the structure of a nondestructive pressure-type parachute rope tension sensor and set the location of the strain gauge patch using the ANSYS Version 12.0 simulation software to obtain a high sensor sensitivity.

In the article [24], similar to [23], a new type of tension sensor is presented according to the needs of measuring the tension of varieties of ropes such as ropes in parachutes. The sensor is designed to have a T-shape structure, so that the rope can cross over the sensor without any damage during measurement.

A measurement method of wire rope tension by transferring wire rope tension measurement to pressure measurement, which improves the measurement safety and avoids the safety hazards of adopting a pull sensor in series with a wire rope, is presented in article [25] by authors G. Xu et al.

The paper [26] describes the structural design of a laboratory device that allows for presenting the operation, simulating work procedures and checking the functionality of the elevator “rope sensors” when equalizing different tensile forces in partial ropes of a rope system of traction elevators. The laboratory device is modified for checking operations of commonly used rope sensors.

The results in the article [27] show that the authors G. Lei et al. proposed compensation improves the accuracy of the real-time measurement system of wire rope tension. Articles [28,29] state that oil pressure sensors are installed on the hydraulic connection device to monitor the tension of wire ropes. The change in acceleration during the operation process of the hoist causes the tension change in each wire rope. It leads to impact on the cylinder of the hydraulic connection device, enlarges the pressure loss and makes the non-linear friction become more complex, which affects the monitoring accuracy.

In order to solve the problem about the tension detection of the multi-hoisting wire rope, the paper [30] designs a dynamic tension detection system of the multi-hoisting wire rope. The system can real-time detect the tension of the rope, alarm to overload, imbalance and other security hazards during the operation and guarantee the safe operation of the elevator. This paper designs a tension sensor for the hoist wire rope.

To improve the accuracy of the tension measurement of bridge wire ropes, research on a magnetic flux sensor based on the magneto-elastic effect and its application in measuring the tension of bridge wire ropes is presented in [31]. A device composed of a magnetic flux sensor and other signal processing circuits is designed, and it can be used to measure the tension on the wire ropes precisely and enduringly by testing the magnetic flux through the sensor without causing damage on wire ropes.

The article [32] contains mathematical formulas that are used for calculations of the necessary adjustment of friction pulley radii aimed at equalizing forces in the individual ropes of the hoisting system.

## 2. Materials and Methods

**Figure 2:**
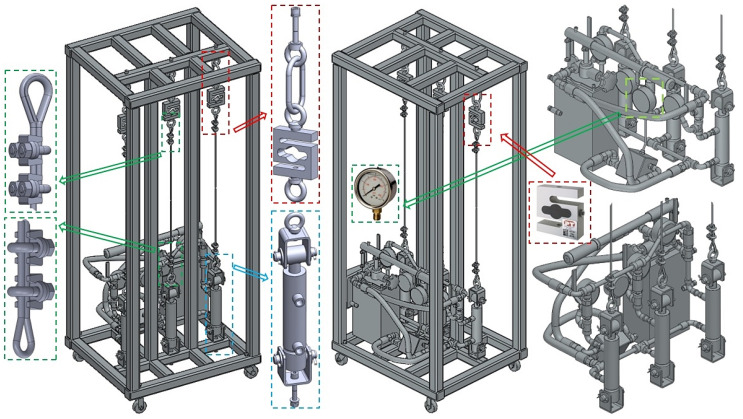
Structural design of laboratory equipment created in SolidWorks^®^ Premium SP 5.0.

The structural design of the laboratory equipment intended for the presentation and simulation of setting the same magnitudes of tensile forces in three cross-sections (n_r_ = 3, see Figure 1) of steel ropes was created in the environment of SolidWorks^®^ Premium SP 5.0, see Figure 2, at the Department of Machine and Industrial Design, Faculty of Mechanical Engineering, VSB-Technical University of Ostrava.

**Figure 3:**
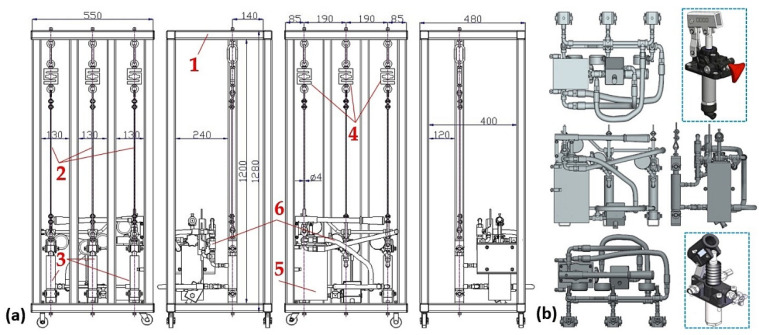
Laboratory equipment (**a**) basic dimensions and components and (**b**) hydraulic circuit. 1—steel structure; 2—steel rope; 3—hydraulic cylinder; 4—tensometric force sensor; 5—manual hydraulic pump; 6—hydraulic circuit.

The laboratory equipment, which allows for setting the same amount of tensile force in the individual cross-sections of the ropes by means of hydraulic oil pressure, consists of a welded (from closed profiles of a square 40 mm × 40 mm cross-section with a wall thickness of 1.5 mm) steel structure 1; see Figure 3a. The hydraulic oil (max. pressure 300 bar = 30 MPa) is distributed by the manual hydraulic pump 5 [33] with a tank of 3 dm^3^ through the designed pipeline of the hydraulic circuit 6 (see Figure 3b) to the double-acting hydraulic cylinders 3 [34].

It is possible to supply hydraulic oil with a working pressure p_p_ = 180 bar = 18 MPa (maximum pressure p_max_ = 250 bar = 25 MPa) to hydraulic cylinders 3 [34] (inner diameter d_c_ = 32 mm, piston rod diameter d_p_ = 20 mm, piston rod stroke h_hp_ = 50 mm). The maximum pressure force F_Rp_ [N] and the pulling force F_R_ [N] that can be derived from the hydraulic cylinder 3 with the calculated cross-section S_c_ [m^2^] and S_hc_ [m^2^], see (2), is given by Relation (3). The cross-section of the hydraulic cylinder in the space above the piston is expressed as S_c_ [m^2^], and the cross-section of the hydraulic cylinder in the space below the cylinder piston is S_hc_ [m^2^].

(2) $$S_{c}=\frac{\pi }{4}\cdot d_{c}^{2}=\frac{\pi }{4}\cdot 32^{2}=804{.2\;\mathrm{mm}}^{2}{;\;S}_{\mathrm{hc}}{=S}_{c}{\;-\;S}_{p}=\frac{\pi }{4}\cdot \left(d_{c}^{2}{\;-\;d}_{p}^{2}\right)=\frac{\pi }{4}\cdot \left(32^{2}{\;-\;20}^{2}\right)=490{.1\;\mathrm{mm}}^{2},$$

(3) $$F_{\mathrm{Rp}}{=p}_{p}{.\;S}_{c}=18\cdot 10^{6}\cdot 8.04\cdot 10^{-4}=\;14476{.5\;N;\;F}_{R}{=p}_{p}{.\;S}_{\mathrm{hc}}=18\cdot 10^{6}\cdot 4.9\cdot 10^{-4}=\;8821.6\;N,$$

On one side, the ends of the steel ropes 2 with a diameter d_r_ = 4 mm of construction 6 × 19M—WSC [35,36] are attached to the piston rods of the hydraulic cylinders 3. The other ends of the steel ropes 2 are attached to the tensometric force sensors 4 [37] with a measuring range of 0 ÷ 2450 N.

**Figure 4:**
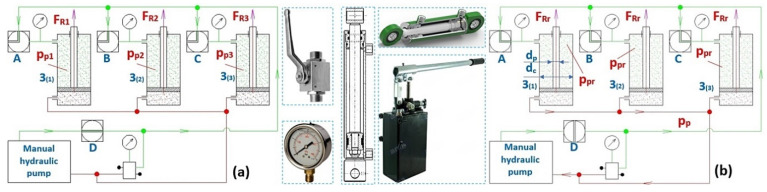
Hydraulic oil pressure in the spaces under the hydraulic cylinder pistons (**a**) of different magnitudes p_p1_ ≠ p_p2_ ≠ p_p3_ [Pa] and (**b**) of the same magnitude p_p1_ = p_p2_ = p_p3_ = p_pr_ [Pa].

Assuming that valves A, B and C supplying/extracting hydraulic oil to the space under the pistons of the hydraulic cylinders 3_(i)_ are closed, see Figure 4a, where i (=1 ÷ 3) is the number of load-bearing ropes, and assuming the forces F_Ri_ [N] in the ropes differ, a hydraulic oil pressure of different magnitudes p_pi_ [Pa] is generated in the space under the pistons of individual hydraulic cylinders 3_(i)_; see (4).

(4) $$p_{p1}=\frac{F_{R1}}{S_{hc}}\;[\mathrm{Pa}],\;p_{p2}=\frac{F_{R2}}{S_{hc}}\;[\mathrm{Pa}],\;p_{p3}=\frac{F_{R3}}{S_{hc}}\;[\mathrm{Pa}],$$

To equalize the different pressures p_pi_ [Pa] of the hydraulic oil in the spaces under the hydraulic cylinder pistons 3_(i)_ to the same pressure value p_pr_ [Pa], see Figure 4b, it is necessary to close valve D of the hydraulic circuit first. In individual hydraulic cylinders 3_(i)_ with hydraulic oil pressure p_pi_ [Pa], there is a certain volume of hydraulic oil V_i_ [m^3^] in the space under the pistons, and in the hydraulic pipe with hydraulic oil pressure p_p_ [Pa], there is a volume of hydraulic oil V_p_ [m^3^]. The maximum possible volume V_M_ [m^3^] of hydraulic oil in the space under the piston of the hydraulic cylinder 3_(i)_ (at the maximum displacement of the piston rod h_hp_ [mm] from the body of the hydraulic cylinder) can be expressed according to Relation (5).

(5) $$V_{M}{=S}_{\mathrm{hc}}\cdot h_{\mathrm{hp}}=4.9\cdot 10^{-4}\cdot 50\cdot 10^{-3}=2.5\cdot 10^{-5}{\;m}^{3},$$

After closing valve D of the laboratory equipment and gradually opening valves A, B and C, the relative pressures p_pi_ [Pa] of the hydraulic oil under the pistons of the hydraulic cylinders 3_(i)_ stabilize at the same value p_pr_ [Pa] (6).

(6) $$p_{\mathrm{pr}}=\frac{p_{p1}\cdot V_{1}{+p}_{p2}\cdot V_{2}{+p}_{p3}\cdot V_{3}+p_{p}{.V}_{p}}{V_{1}{+V}_{2}{+V}_{3}{+V}_{p}}\;[\mathrm{Pa}],$$

**Figure 5:**
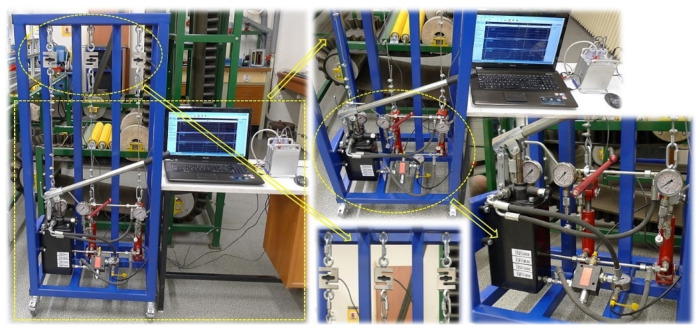
Laboratory equipment simulating method of hydraulic equalization of pulling force values in ropes.

The implemented laboratory equipment, which was built in the Laboratory of Research and Testing, Department of Machine and Industrial Design, Faculty of Mechanical Engineering, VSB-Technical University of Ostrava, is presented in Figure 5.

**Figure 6:**
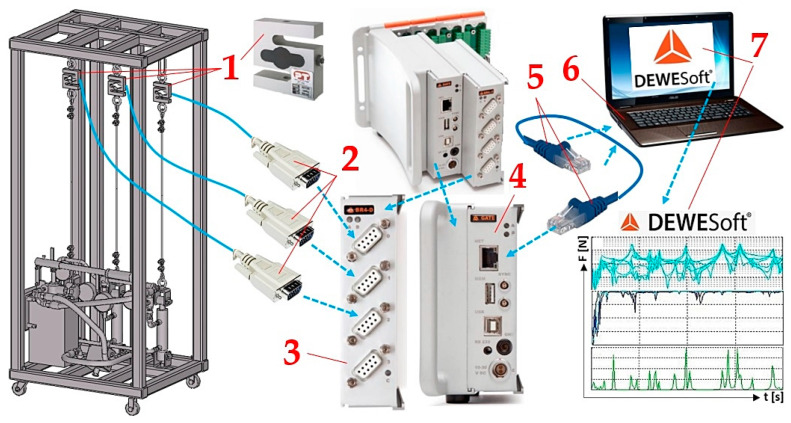
Measuring chain—a sequence of connected instruments and devices enabling the detection and processing of measured signals. 1—tensometric force sensor; 2—D-sub DE-9 connector; 3—DS NET BR4 module; 4—DS GATE module; 5—Ethernet cable; 6—ASUS K72JR-TY131; 7—DEWESoft^®^ X2 SP5.

A system (the so-called measurement chain) created from a sequence of devices and other instruments connected in a way which makes it possible to process the measurement signal from the input measured quantity up to obtaining the output value is shown in Figure 6. The plugs of the “D-sub DE-9” connectors 2, which terminate the wires of the three tensometric force sensors 1 [37], are inserted into the sockets of the DS NET BR4 module 3 [37]. The DS GATE 4 [38] module is connected to the PC (ASUS K72JR-TY131 (Taipei, Taiwan)) 6, on which the DEWESoft X2 SP5 7 (Gabrsko, Slovenia) [39] software is installed, through the RJ45 connectors 5 of the network cable.

## 3. Results

Experimental measurements performed on laboratory equipment, see Figure 5, were carried out in several independent experiments.

**Figure 7:**
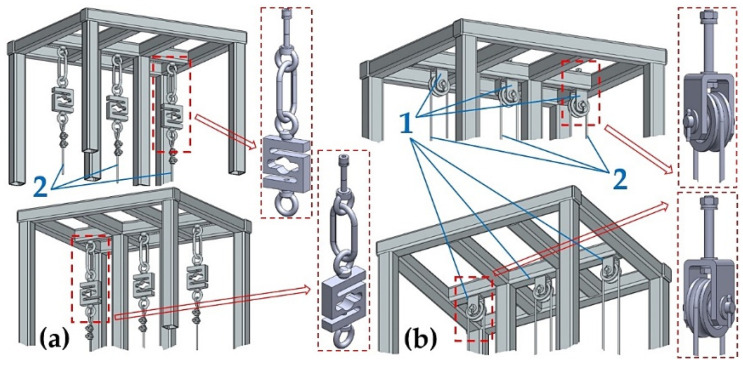
Steel ropes (**a**) attached to the upper part, (**b**) guided by the grooves of the pulleys of the laboratory equipment.

The free ends of three steel ropes 1 of finite length were attached to the upper part, see Figure 7a, of the steel frame of the laboratory equipment.

**Figure 8:**
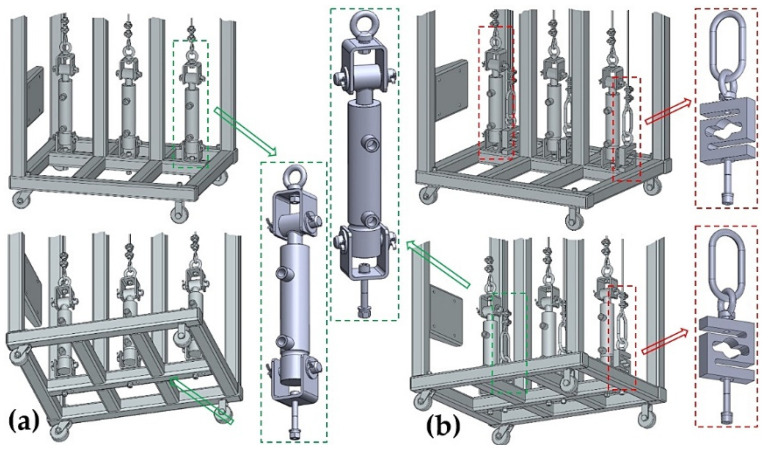
Ends of the steel ropes attached (**a**) to the upper part and (**b**) to the lower part of the laboratory equipment.

In the second variant, the ends of three double-length steel ropes were attached to the lower part, see Figure 8b, of the steel frame of the laboratory equipment.

In both cases, the attachment of the ends of the ropes (to the upper part Figure 7a or to the lower part Figure 8b of the laboratory equipment) exerts the same amount of pulling force on the steel rope assuming an identical insertion of the piston rod into the body of the hydraulic cylinder, i.e., at the same hydraulic oil pressure under the hydraulic cylinder piston.

### 3.1. The Magnitude of the Resulting Hydraulic Oil Pressure Is Affected by the Different Values of the Hydraulic Oil Pressures in the Hydraulic Cylinders and the Pressure in the Supply Pipe

**Figure 9:**
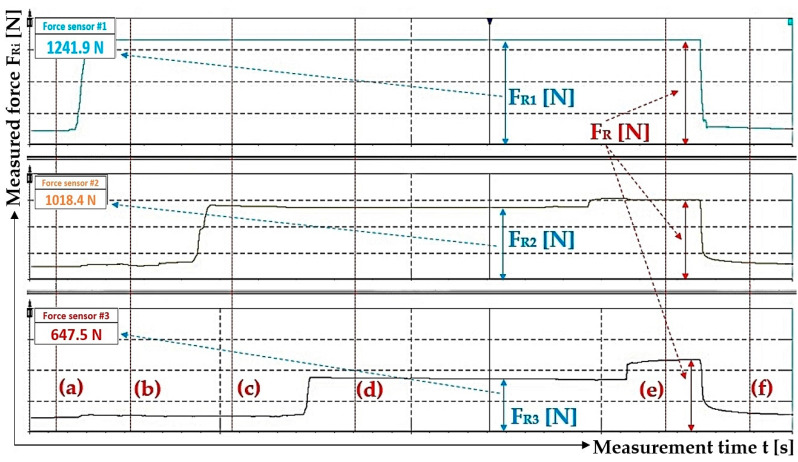
Time recording of measured pulling forces in ropes by tensometric force sensors on laboratory equipment.

The measurements were carried out under the same technical conditions. Tensometric force sensors 4 (Figure 3) were calibrated with a load of known weight before the experimental tests were performed on the laboratory equipment (Figure 5). At the beginning of the measurements, the piston rods were fully extended from the bodies of all hydraulic cylinders 3, whereby the tensometric force sensors 4 were loaded only by the weight of the freely suspended ropes 2. In Figure 9, this state is designated as (a).

The hydraulic oil was supplied under a pressure of p_p1_ [Pa] by the manual hydraulic pump lever, with valves D and A open (see Figure 4) and valves B and C closed, under the hydraulic cylinder piston 3_(1)_. The pressure p_p1_ [Pa] of the hydraulic oil acting on the piston of the hydraulic cylinder 3_(1)_ pushed the piston rod into the body of the hydraulic cylinder 3_(1)_, thereby generating a pulling force F_R1_ [N] of a certain magnitude in the steel rope 2, which was detected by the tensometric force sensor 4. In Figure 9, this state is designated as (b).

After valve A was closed (with valve D open and valves B and C closed), valve B was opened and hydraulic oil was fed into the space under the piston of the hydraulic cylinder 3_(2)_ under a pressure of p_p2_ ≠ p_p1_ [Pa], then valve B was closed. The pulling force F_R2_ [N] in the steel rope 2 detected by the tensometric force sensor 4 is directly proportional to the size of the applied pressure p_p2_ [Pa] of the hydraulic oil under the piston of the hydraulic cylinder 3_(2)_. In Figure 9, this state is designated as (c).

With valve D and C open (and valves A and B closed), hydraulic oil was pumped under pressure p_p3_ ≠ p_p2_ ≠ p_p1_ [Pa] into the space under the hydraulic cylinder piston 3_(3)_. When the pressure p_p3_ [Pa] was reached in the space under the piston of the hydraulic cylinder 3_(3)_, valves C and D were simultaneously closed. The pulling force F_R3_ [N] in the steel rope 2 detected by the tensometric force sensor 4 is directly proportional to the magnitude of the supplied pressure p_p3_ [Pa] of hydraulic oil under the hydraulic cylinder piston 3_(3)_. In Figure 9, this state is designated as (d).

When valves A, B, C and D are closed, hydraulic oil pressure p_p_ = p_p3_ [Pa] is present in the supply pipe.

With valve D closed and hydraulic oil pressure in the supply pipe of the hydraulic circuit p_p_ [Pa], valves A, B and C were gradually opened. Valve A was opened first. The different pressure values p_p1_ [Pa] and p_p_ = p_p3_ [Pa] (with valves B, C and D closed) stabilized at the same pressure of p_p1p_ [Pa]. The magnitude of the pressure p_p1p_ [Pa] is dependent on whether the pressure p_p1_ [Pa] is less or greater than the pressure p_p_ [Pa] and also on the volumes V_1_ [m^3^] and V_p_ [m^3^]. During experimental measurements, it was not possible to accurately measure the volumes of hydraulic oil in the spaces above the piston V_i_ [m^3^] for all hydraulic cylinders nor the volume V_p_ [N] of hydraulic oil in the pipes.

Assuming that the volume of hydraulic oil under the piston V_1_ [m^3^] of the hydraulic cylinder 3_(1)_ is the same as the amount of hydraulic oil in the pipe V_p_ [m^3^] of the laboratory equipment, then the resulting pressure is p_p1p_ = 0.5· (p_p1_ + p_p_) [Pa]. If the volume of hydraulic oil under the piston of the hydraulic cylinder V_1_ [m^3^] is greater, less or equal to the volume of hydraulic oil in the hydraulic pipe V_p_ [m^3^] and if pressure p_p1_ = p_p_ [Pa], then the resulting pressure is p_p1p_ = p_p1_ [Pa].

**Figure 10:**
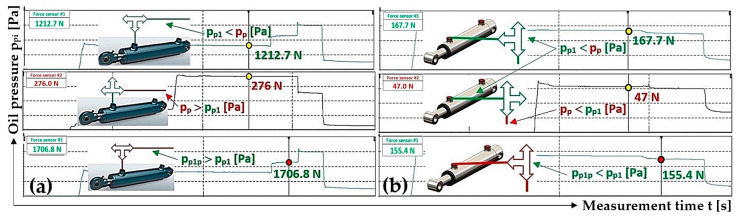
Supply pipe pressure p_p_ = p_p3_ [N] (**a**) greater than pressure p_p1_ [N] and (**b**) less than pressure p_p1_ [N].

The magnitude of the resulting pressure p_p1p_ [Pa] is greater than p_p1_ [Pa] if p_p_ > p_p1_ [Pa]; see Figure 10a. The magnitude of the pressure p_p1p_ [Pa] is less than p_p1_ [Pa] if p_p_ < p_p1_ [Pa]; see Figure 10b.

In the next steps, first valve B and then valve C were opened (while valve D was closed). The pressures in the spaces above the pistons in all three hydraulic cylinders (when valves A, B and C are open) as well as the pressure in the supply pipe of the hydraulic circuit (when valve D is closed) stabilize at the same pressure p_pr_ [Pa], the theoretical magnitude of which can be described by Relation (6). In Figure 9, this state is designated as (e).

After valve D is opened, the hydraulic oil moves to the tank of the manual hydraulic pump, so that the pressure of the hydraulic oil in the supply pipe of the hydraulic circuit and in the space under the pistons of all hydraulic cylinders is zero (takes on the value of hydrostatic pressure). In Figure 9, this condition is designated as (f).

Table 1 presents different initial values of pulling forces F_Ri_ [N] in ropes 2, measured using tensometric force sensors 4, see Figure 3, which were generated by the pressure of the oil supplied by the hydraulic pump into the space under the pistons of the hydraulic cylinders 3_(i)_. The measured pulling forces F_Ri_ [N] in ropes 2, during the gradual filling (first cylinder 3_(1)_ and last cylinder 3_(3)_) of hydraulic oil into the space under the pistons of hydraulic cylinders 3_(i)_ of the laboratory equipment, were used to calculate the pressures p_pi_ [Pa] of hydraulic oil under the piston of the hydraulic cylinder 3_(i)_.

**Figure 11:**
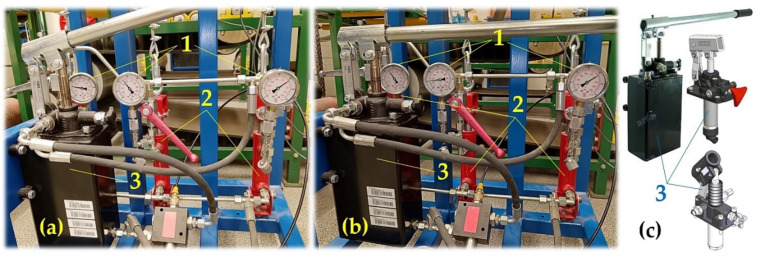
(**a**,**b**) Hydraulic circuit of laboratory equipment and (**c**) manual hydraulic pump. 1—manometer; 2—double-acting hydraulic cylinder; 3—manual hydraulic pump.

The calculated pressures p_pi_ [Pa] were verified with the measured (using manometers; see Figure 11) hydraulic oil pressures under the pistons of hydraulic cylinders 3_(i)_. The manometer detected pressures in the unit of bar = 1·10^5^ Pa (0.1 MPa).

**Table 1:** The pulling force in the rope and the pressure of the hydraulic oil in the space under the hydraulic cylinder piston and in the supply pipe.

n	F_R1_	F_R2_	F_R3_	p_p1_	p_p2_	p_p3_ = p_p_
	[N]			[10^6^·Pa]		
1	786.9 ^1^	1170.2 ^1^	2090.4 ^1^	1.61 ^2^	2.39 ^2^	4.27 ^2^
2	1158.7	531.9	2127.8	2.36 ^3^	1.09 ^3^	4.34 ^3^
3	696.1	1170.2	2203.5	1.42	2.39	4.50

^1^ see Figure 12a; ^2^ see Figure 11a; ^3^ see Figure 11b.

**Figure 12:**
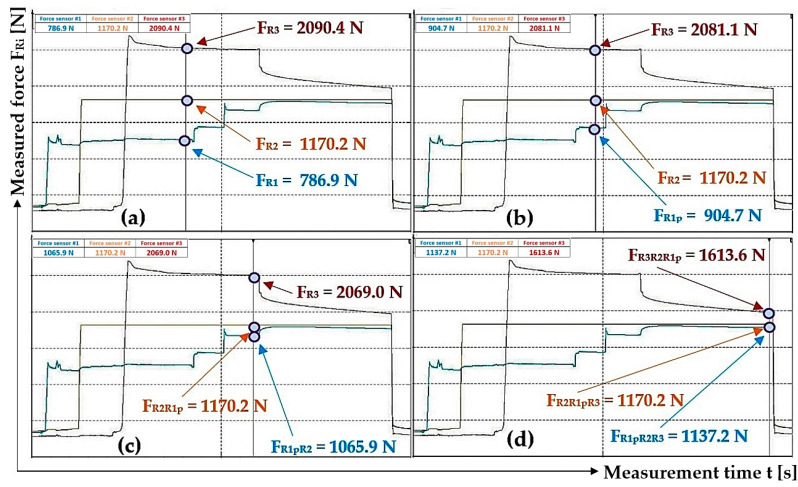
Measured pulling forces in ropes with (**a**) valves A to D closed, (**b**) valve A open and valves B to D closed, (**c**) valves A and B closed and valves C and D closed and (**d**) valves A to C open and valve D closed.

Figure 12a presents the values of the measured pulling forces, which were generated by the pressure of the hydraulic oil supplied under the pistons of the hydraulic cylinders during the gradual opening and closing of valves A, B and C while valve D is open; see Figure 4.

Figure 12b shows the value of the detected force F_R1p_ [N] (in the rope above the hydraulic cylinder 3_(1)_) which was measured by the tensometric force sensor (with valves B, C and D closed) at the moment when valve A was opened. At this moment, the pressure p_p1_ [Pa] of the hydraulic oil under the piston of the hydraulic cylinder 3_(1)_ equalized with the pressure of the hydraulic oil in the pipe p_p_ [Pa] to the value of p_p1p_ [Pa], and the pulling force F_R1p_ was acting in the rope above the cylinder 3_(1)_ [N].

Table 2 lists the values of the forces detected in the ropes 2 by the tensometric force sensors 4 at the moment when valves A, B and C were gradually opened while valve D was left closed.

**Table 2:** The pulling force in the ropes during the gradual opening of valves A, B and C supplying hydraulic oil to the space under the pistons of the hydraulic cylinders.

n	F_R1p_	F_R1pR2_	F_R2R1p_	F_R1pR2R3_	F_R2R1pR3_	F_R3R2R1p_	p_p3p2p1p_ = p_pr_
	[N]						[10^6^·Pa]
1	904.7 ^1^	1065.9 ^2^	1170.2 ^2^	1137.2 ^3^	1170.2 ^3^	1613.6 ^3^	3.29
2	1158.7	1158.7	806.1	1158.7	1009.4	1404.1	2.86
3	878.9	1170.2	2175.5	1052.5	1170.2	1623.7	3.31

^1^ see Figure 12b; ^2^ see Figure 12c; ^3^ see Figure 12d.

Figure 12c shows the values of the detected forces F_R1pR2_ [N] (in the rope above the hydraulic cylinder 3_(1)_) and F_R2R1p_ [N] (in the rope above the hydraulic cylinder 3_(2)_), which were measured by the tensometric force sensors when valve B was opened (with valves C and D closed and valve A open). At this moment, the pressure p_p2_ [Pa] of the hydraulic oil under the piston of the hydraulic cylinder 3_(2)_ equalized with the pressure of the hydraulic oil in the pipe p_p1p_ [Pa] (as well as in the space under the piston of the hydraulic cylinder 3_(1)_) to the value p_p2p1p_ [Pa] and pulling forces F_R1pR2_ [N] (in the rope above the hydraulic cylinder 3_(1)_) and F_R2R1p_ [N] (in the rope above the hydraulic cylinder 3_(2)_) were measured in the ropes.

Figure 12d shows the values of the detected forces in the ropes above the hydraulic cylinders 3_(i)_ that were measured by the tensometric force sensors at the moment when valve C was opened (with valve D closed and valves A and B open). At this moment, the pressure p_p3_ [Pa] of the hydraulic oil under the piston of the hydraulic cylinder 3_(3)_ equalized with the pressure of the hydraulic oil in the pipe p_p2p1p_ [Pa] to the value p_p3p2p1p_ [Pa], and pulling forces F_R1pR2R3_ [N] (in the rope above hydraulic cylinder 3_(1)_), F_R2R1pR3_ [N] (in the rope above hydraulic cylinder 3_(2)_) and F_R3R1pR2_ [N] (in the rope above hydraulic cylinder 3_(3)_) were measured in the ropes.

By analyzing the individual phases of the measurements, see Figure 12b–d and Table 2, on the laboratory device (see Figure 5), it can be observed that when valves A to C are gradually opened, the measured pulling forces in the ropes are not completely equalized. The pressure p_p1_ [Pa] (pulling force F_R1_ [N]), when opening valve A, should equalize to the same magnitude as the hydraulic oil pressure in the hydraulic pipe. The magnitude of pressure p_p1p_ [Pa] depends on the magnitudes of the volumes V_1_ [m^3^] and V_p_ [m^3^] and the magnitudes of the initial pressures p_p1_ [Pa] and p_p_ [Pa]. According to Relation (7), it is possible to calculate the theoretical pressure p_p1p_ [Pa] under the piston of the hydraulic cylinder and the pressure in the pipe when valve A is open (and valves B, C and D are closed).

(7) $$p_{p1p}=\frac{p_{p_{1}}\cdot V_{1}{+p}_{p}{.V}_{p}}{V_{1}{+V}_{p}}{\;[\mathrm{Pa}],\;p}_{p1\mathrm{pp}2}{=p}_{p1p}+\frac{p_{p2}\cdot V_{2}}{V_{2}}=\frac{p_{p1}\cdot V_{1}{+p}_{p2}\cdot V_{2}{+p}_{p}{.V}_{p}}{V_{1}{+V}_{2}{+V}_{p}}\;[\mathrm{Pa}]$$

The volume of hydraulic oil in the hydraulic pipe V_p_ [m^3^] is constant and does not change in the laboratory equipment during all experimental measurements. The volumes V_i_ [m^3^] of hydraulic oil under the pistons of the hydraulic cylinders are different for each measurement performed. Their values depend on the magnitude of the supplied hydraulic oil pressure under the piston of the respective hydraulic cylinder (3; see Figure 3), i.e., on how far the piston rod is inserted into the hydraulic cylinder body. Due to the different and unknown volumes V_i_ [m^3^], Relation (7) does not allow for the calculation of the theoretical magnitude of the hydraulic oil pressures in the hydraulic circuit pipeline when opening valves A and B without the knowledge of the volumes V_i_ [m^3^].

### 3.2. The Magnitude of the Resulting Pressure of the Hydraulic Oil Affected by the Change in the Different Magnitudes of the Pulling Forces in the Ropes

The hydraulic pump (Figure 11c) was used to supply hydraulic oil with a pressure of p_p_ [Pa] through the hydraulic pipe (when valves A, B and C of all three hydraulic cylinders 3_(i)_ are open; see Figure 4) into the space under the pistons of hydraulic cylinders 3_(i)_. The pressure of the hydraulic oil p_pi_ [Pa] under the pistons of the hydraulic cylinders 3_(i)_ caused the piston rods to be inserted into the bodies of the hydraulic cylinders 3_(i)_ and generated the pulling forces in ropes F_Ri_ [N], whose magnitudes were detected by the tensometric force sensors 4 (Figure 3) [37]. After valve D was closed, the pressure of the hydraulic oil in the hydraulic pipe and the pressure under the pistons of the hydraulic cylinders 3_(i)_ was p_p_ [Pa], which can be expressed according to Relation (4), provided that the values of forces F_Ri_ [N], which were detected by tensometric force sensors, are known.

When valves A, B and C of the hydraulic cylinders 3_(i)_ are closed (provided that valve D is closed) and the screws that mechanically attach the suspension nuts of the tensometric force sensors 4 (Figure 3) to the steel frame of the laboratory equipment 1 are gradually tightened, the pulling forces F_Ri_ [N] in the ropes 2 differed. The maximum pulling force F_Ds_ [N], which could be generated in the ropes 2, is given by the permissible load m_Ds_ = 250 kg (F_Ds_ = 2.45 kN) of the tensometric force sensor 4.

The magnitudes of pulling forces F_Ri_ [N] measured by tensometric force sensors 1, see Figure 6, were recorded by modules 3 and 4 of the DEWESoft DS-NET measuring apparatus and displayed on the PC monitor 6 in the environment of DEWESoft^®^ X2 SP5 7; see Table 3.

From the measured pulling forces F_Ri_ [N], the pressures p_pi_ [Pa] in the hydraulic cylinders 3_(i)_ were calculated according to Relation (4) (using the known cross-section S_hc_ [m^2^] of the hydraulic cylinder surface).

**Figure 13:**
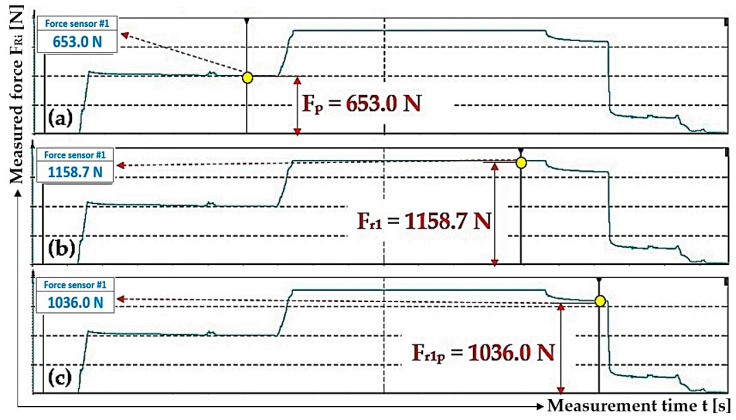
The pulling force in the rope, with valves B and C of the hydraulic circuit closed, when (**a**) pumping oil under the piston of the hydraulic cylinder with valve A open, (**b**) applying the pulling force in the rope with valve A closed and (**c**) opening valve A.

When valve A of the hydraulic cylinder 3_(1)_ is opened (and when valves B, C and D are closed), the pressure p_p1_ [Pa] of the hydraulic oil under the piston of the hydraulic cylinder 3_(1)_ (at volume V_1_ [m^3^]) and the pressure p_p_ [Pa] of the hydraulic oil in the supply pipe (at volume V_p_ [m^3^]) of the hydraulic circuit equalized to the pressure value p_p1p_ [Pa]; see Figure 13.

The pressure of the hydraulic oil p_i_ [Pa] in the spaces under the pistons of the hydraulic cylinders 3_(i)_ generates pulling forces F_Ri_ [N] in the i-th steel rope, and their magnitudes are detected by tensometric force sensors 4 (see Figure 3). The hydraulic oil pressures p_i_ [Pa], as recorded in Table 4, were calculated from the recorded values of pulling forces F_Ri_ [N] according to Relation (4).

**Table 4:** The pulling force in the rope and the pressure of the hydraulic oil in the space under the hydraulic cylinder piston and in the supply pipe.

n	F_p1_	F_p2_	F_p3_	F_R1_	F_R2_	F_R3_	p_p_	p_p1_	p_p2_	p_p3_
	[N]						[MPa]			
1	285.9 ^1^	318.6 ^1^	297.2 ^1^	642.3 ^2^	1170.2 ^2^	1921.5 ^2^	0.58	1.31	2.39	3.92
2	606.3	615.6	618.0	1034.5	1170.2	1677.8	2.24	1.26	2.39	3.42
3	534.5	556.5	503.4	1104.0	1170.2	1816.4	1.09	2.25	2.39	3.71

^1^ see Figure 14a; ^2^ see Figure 14d.

**Figure 14:**
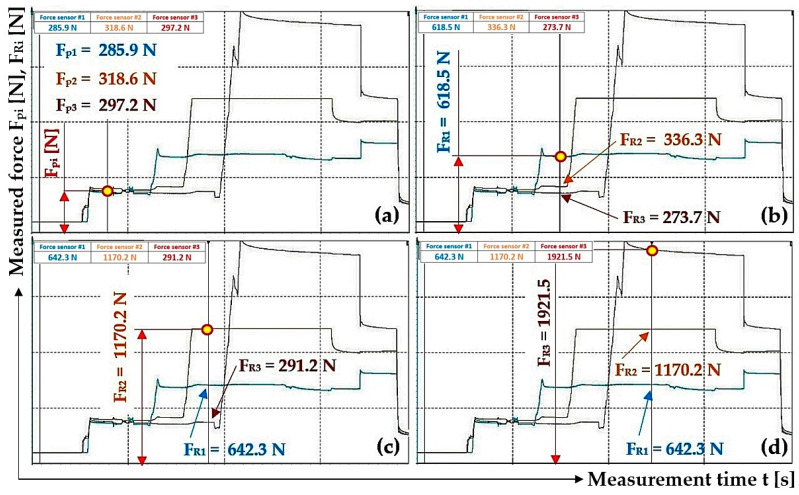
Detected pulling forces in the ropes, generated by applying hydraulic oil pressure under the hydraulic cylinder pistons: (**a**) valves A to D open; (**b**) valves A and D open, valves B and C closed; (**c**) valves B and D open, valves A and C closed and (**d**) valves C and D open, valves A and B closed.

**Figure 15:**
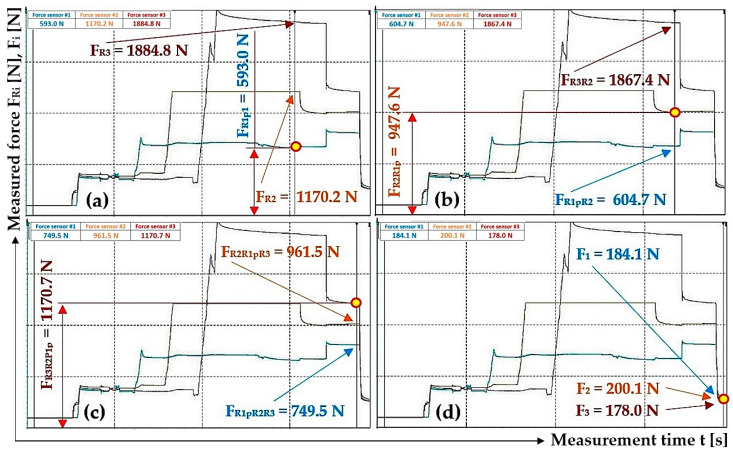
The detected pulling forces in the ropes, generated by applying hydraulic oil pressure under the hydraulic cylinder pistons: (**a**) valve A open, valves B to D closed; (**b**) valve B open, valves C and D closed; (**c**) valve C open, valve D closed and (**d**) valve D open.

By analyzing Figure 15a–c, it can be observed that when valves A, B and C are gradually opened, the pulling forces (detected by tensometric force sensors [36]) acting in the ropes, as well as the hydraulic oil pressures in the spaces under the pistons of the hydraulic cylinders, did not equalize to the same value. The pulling forces in all three ropes equalized to approximately the same value only after valve D was opened; see Figure 15d.

**Figure 16:**
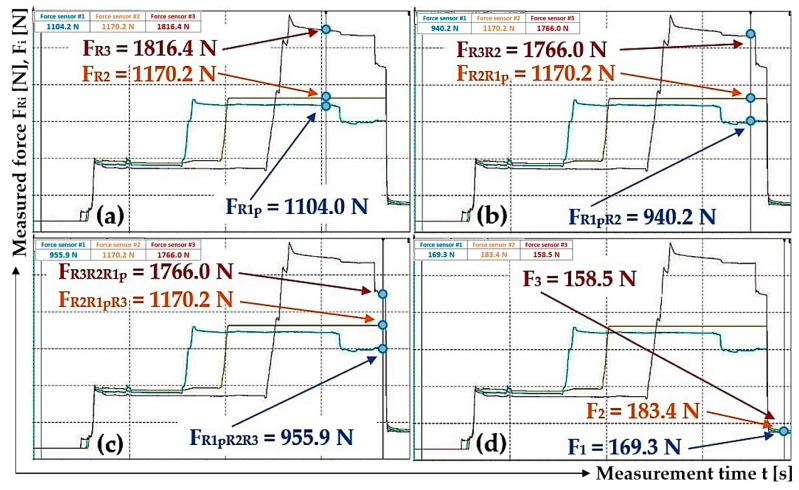
The detected pulling forces in the ropes, generated by applying hydraulic oil pressure under the hydraulic cylinder pistons: (**a**) valve A open, valves B to D closed; (**b**) valve B open, valves C and D closed; (**c**) valve C open, valve D closed and (**d**) valve D open.

The different values of pulling forces, see Figure 16c, can be explained by the fact that the spaces above the pistons of the hydraulic cylinders are interconnected by a closed and sealed hydraulic pipe. When the piston rods are inserted into the hydraulic cylinders, the contact surfaces of the seals (polyurethane) of the pistons with the inner surface of the hydraulic cylinders create friction between these contact surfaces, which acts as a resistance force when the piston rods are inserted into the hydraulic cylinders.

## 4. Discussion

The implemented laboratory equipment, as described in this article, can be used in practice for the continuous equalization of pulling forces in elevator ropes but especially in mining machines with friction discs, where winding ropes of significantly longer lengths than the load-bearing ropes of elevators are used. The advantage of the presented laboratory equipment is that it can gradually reduce the pulling force in one rope if it is larger than the pulling forces in the neighboring ropes of the multi-rope system.

Brugg’s Rope Load Equalizer (RLE) [14] is a hydraulic device that uses hydraulic fluid pumped through an interconnected system of hoses into hydraulic cylinders to create a tensile force of the same magnitude in all elevator rope fastening sockets. This device is installed on a specific traction elevator by an authorized person only when it is required to set or verify that the load from the weight of the cage or counterweight is evenly distributed over the designed number of load-bearing ropes.

Mechanical devices [11,12,22] are also installed, similarly to device [14], on a particular traction elevator when it is required to set or verify that the load from the weight of the cage or counterweight is evenly distributed over the designed number of load-bearing ropes.

Figure 12d shows the measured values of pulling forces that were detected by tensometric force sensors with valves A to C open and valve D closed in the laboratory device (Figure 4). This condition (valves A to C open, valve D closed) describes the case when the hydraulic oil pressure is the same in the spaces under the pistons of all hydraulic cylinders as well as in the hydraulic pipeline. With the same amount of pressure (valves A to C open), the same pulling force should act in all three ropes. However, the measured values of pulling forces in individual ropes are not completely identical. The highest measured value of a pulling force is 1613.6 N and the lowest is 1137.2 N; see Table 2. The highest measured value of a pulling force is 41.9% higher than the lowest measured value.

The reason for the difference in the pulling forces, which were detected by tensometric force sensors (see Figure 12d) and which act in the individual ropes when valves A to C are open, was attributed to the increasing hydraulic oil pressure in the spaces above the pistons of the individual hydraulic cylinders when the piston rods were inserted into the bodies of the hydraulic cylinders by the pressure of the hydraulic oil p_pi_ [Pa] supplied under the pistons of the hydraulic cylinders. It was assumed that the pressure of the hydraulic oil in the closed pipeline as well as the pressure in the spaces above the pistons of the hydraulic cylinders acts as a resistance force when the pistons are pushed into the hydraulic cylinders. This assumption was disproved by the fact that only hydrostatic pressure acts in the spaces above the pistons, as the spaces above the pistons are connected by a hydraulic pipe that flows into the tank of the manual hydraulic pump, which is vented, i.e., no pressure other than hydrostatic pressure acts on the hydraulic oil.

Figure 15c and Figure 16c also present the case when the hydraulic oil pressure in the spaces under the pistons of all hydraulic cylinders is the same as in the hydraulic pipeline. The highest measured value of a pulling force, see Figure 15c and Table 5, is 1170.7 N and the lowest is 749.5 N. The highest measured pulling force value is 1.56 times (56.2%) higher than the lowest measured value. The highest measured value of the pulling force, Figure 16c, is 1766.0 N and the lowest is 955.9 N; see Table 5. The highest measured value of the pulling force is 84.7% higher than the lowest measured value.

After opening valve D, which reduces the pressure of the hydraulic oil in the hydraulic pipeline and the pressure in the spaces under the pistons of the hydraulic cylinders to the value of the atmospheric pressure, the values of the pulling forces in the ropes acquire approximately the same magnitude; see Figure 15d. The highest measured value of the pulling force, Figure 15d, is 200.1 N and the lowest is 178.0 N; see Table 5. The highest measured value of the pulling force is 12.4% (1.12 times) higher than the lowest measured value. The highest measured value of the pulling force, Figure 16d, is 183.4 N and the lowest is 158.5 N; see Table 5. The highest measured value of the pulling force is 1.16 times (15.7%) higher than the lowest measured value.

According to [40], it can be stated that the efficiency of a linear hydraulic motor reaches values of 94% to 96%. The difference in the measured values of pulling forces, see Figure 12d, Figure 15c and Figure 16c, on the laboratory equipment (Figure 5), acting in individual ropes when valves A to C are open, is a consequence of the total resistance force, which can be expressed for a specific hydraulic cylinder as the sum of the frictional forces in the contact surface of the piston seal with the inner cylindrical surface of the hydraulic cylinder body and of the rod seal with the cap.

The difference between the highest and the lowest measured pulling force in the ropes, see Figure 12d, is F_dif_ = 1613.6 − 1137.2 = 476.4 N. This value corresponds to the hydraulic oil pressure under the hydraulic cylinder piston (cross-section S_hc_ [m^2^] (2)) p = F_dif_/S_hc_ = 476.4/4.9·10^−4^ = 9.7·105 Pa. With the calculated mean value p_r_ = 2.67 MPa of the actual pressures under the pistons of the hydraulic cylinders (p_p1_ = 3.29 MPa, p_p2_ = 2.39 MPa and p_p3_ = 2.32 MPa), the pressure p = 1 MPa becomes 37.5% of the value p_r_ = 2.67 MPa.

The difference between the highest and lowest measured pulling force in the ropes, see Figure 15c, is F_dif_ = 1170.7 − 749.5 = 421.2 N. This value corresponds to the hydraulic oil pressure under the hydraulic cylinder piston p = F_dif_/S_hc_ = 421.2/4.9·10^−4^ = 8.6·10^5^ Pa.

The difference between the highest and lowest measured pulling force in the ropes, see Figure 16c, is F_dif_ = 1766.0 − 955.9 = 810.1 N. This value corresponds to the hydraulic oil pressure under the hydraulic cylinder piston p = 1.7·10^6^ Pa.

**Figure 17:**
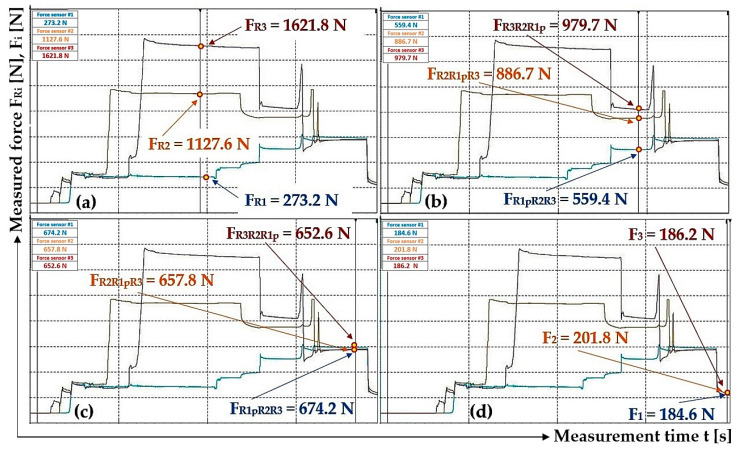
The detected pulling forces in the ropes, generated by applying hydraulic oil pressure under the hydraulic cylinder pistons. (**a**) Valve A to D closed. Opened valves (**b**), (**c**) A to C, valve D closed, and (**d**) A to D.

Figure 17b also presents the case when the hydraulic oil pressure in the spaces under the pistons of all hydraulic cylinders is the same as in the hydraulic pipeline. The highest measured value of the pulling force is 979.7 N and the lowest is 559.4 N. The highest measured value of the pulling force is 1.75 times (75.1%) higher than the lowest measured value.

The highest measured value of the pulling force, see Figure 17c, is 674.2 N and the lowest is 652.6 N. The highest measured pulling force value is 1.03 times (3.3%) higher than the lowest measured value.

**Figure 18:**
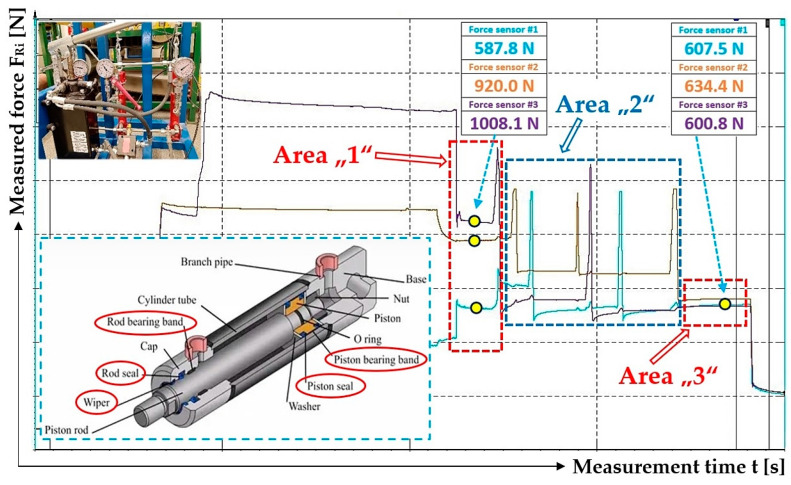
Phases to achieve the same magnitudes of pulling forces in the load-bearing ropes of the laboratory equipment.

The high differences in the values of pulling forces in the ropes (Figure 12d, Figure 15c, Figure 16c and Figure 17b), when valves A to C are open, are caused by resistance forces that prevent the piston rods from being pushed out of the hydraulic cylinder bodies without the action of external forces. The magnitudes of the resistance against the movement of the piston rod in the body of the hydraulic cylinder vary for hydraulic cylinders of different constructions. The amount of resistance against the movement of the piston rod depends on the type of seals used and the clearance (which ensures the tightness of the hydraulic oil pressure) between the seal and the inner cylindrical surface of the hydraulic cylinder body. The phases of experimental measurements carried out in the Laboratory of Research and Testing, Department of Machine and Industrial Design, Faculty of Mechanical Engineering, VSB-Technical University of Ostrava, when valves A to C were opened, proved that due to the resistance forces acting against the movement of piston rods in the bodies of hydraulic cylinders, the different values of pulling forces in the ropes were not equalized to the same value. This phase is represented by Area “1”; see Figure 18. Due to the fact that during the measurements of pulling forces in the ropes, the ropes were static (i.e., they were not carried away by friction in the grooves of the friction discs, as is the case with the friction drives of elevators or mining machines), no external dynamic forces act on the ropes after opening valves A to C.

When additional pulling forces were applied to the ropes (due to the lateral deflection of the ropes with the help of the hand of the operator of the laboratory equipment), see Figure 18 Area “2”, and these overcame the resistance forces in the hydraulic cylinders, the pulling forces in all ropes were equalized to the same values; see Figure 18 Area “3”.

Based on the measurements carried out, it can be stated that in order to achieve the easiest and most accurate hydraulic equalization of the pulling forces in ropes, it is particularly necessary to choose suitable hydraulic cylinders and take into account their operating parameters, which are mainly the tensile/compressive force, operating pressure and structural dimensions. The structural dimensions of the hydraulic cylinder and the hydraulic oil pressure affect the amount of the resistance force required to extend/retract the piston rod into the hydraulic cylinder body. The partial components of the total resistance force required for extending/retracting the piston rod into the body of the hydraulic cylinder are, see Figure 18, rod seal resistance, rod bearing resistance, piston seal resistance, piston bearing resistance and wiper friction resistance.

To achieve an absolutely accurate distribution of the total pulling force among the specified number of load-bearing ropes, it would be necessary for the resistance against the extending/retracting of the piston rod into the body of the hydraulic cylinder to be zero. Due to the fact that friction arises (and cannot be completely eliminated) during the movement of the piston rod in the body of the hydraulic cylinder, it is necessary to limit this friction to the smallest possible extent by using the most suitable type of hydraulic cylinder, so that it is possible to derive the values of pulling forces in partial ropes with only minor deviations.

## 5. Conclusions

The performed measurements made it possible to trace that there is no complete equalization of the different values of the pulling forces in all three ropes (if there are no additional pulling forces in the ropes) (Figure 12d, Figure 15c, Figure 16c and Figure 17b), and this is because of the resistance forces that act when the piston rods are pushed out of the hydraulic cylinder bodies.

In the laboratory equipment, the ropes are static, i.e., they are not in motion as in real mining equipment/elevators, where the traction/load-bearing ropes are carried by the grooves of the friction discs due to friction. As a result of the generated dynamic pulling forces that act in the longitudinal axes of the traction/load-bearing ropes during their movement at lifting speed, and when they pass through the friction discs, these pulling forces in the ropes are transferred to the piston rods of the hydraulic cylinders and reduce or completely eliminate (if they are greater than the resistance against the extension of the piston rod) the magnitude of the frictional force when the piston rod is extended from the hydraulic cylinder body. The absence of dynamic pulling forces in the ropes of the laboratory equipment was replaced by additional pulling forces created by the operator of the laboratory equipment, thus achieving an exact equalization of the pulling forces in all three ropes (Figure 17c and Figure 18).

Several principles are currently known in the consumer market allowing for the subtraction of the acting tensile force in the ropes and eventually offsetting the different tensile force values in the individual ropes.

Well known is the principle of a rope sensor, e.g., [41], measuring the tensile force in the rope using the principle of the bending deformation of the beam loaded with a single force.

A certain limitation in the use of the method for determining the tensile force in the carrier rope with a rope sensor can also be seen in the necessity to have this device mechanically attached on the ropes during the offset of the tensile forces. In a phase of operation, when new ropes are being installed in the elevator system and it is necessary to move the cage within the elevator shaft in order to offset tensile forces in the individual ropes, there is a risk of the collision of the sensors attached to the ropes with the friction disc or other elements in the elevator shaft.

Another significant limitation presents the possibility of damaging the power cables to the tensometric sensors of the rope sensors, when there is a two-way movement of the elevator cage. When the cage is moving within the elevator shaft, then the carrier ropes and thus also the rope sensors, which are mechanically attached to the cross-sections of the ropes, are in motion as well.

The actual value of the tensile force acting on the rope axis is not directly measured by the rope sensor; it is determined proportionally from the normal force exerted, i.e., the force perpendicular to the rope axis. This normal force is a resultant of the components of the acting tensile forces in inclined sections of the measured rope and acts on the central contact member of the rope sensor. The actual value of the tensile force in the rope must therefore be determined by the comparative method. The accuracy of determining the actual tensile force in the rope is affected by the reshaping (deformation) of the rope sensor body and depends on the angle of inclination of the rope section and the distance of the gripping points of all contact points.

From the experimental measurements carried out (presented in Section 3 Results), it follows that by using the pressure of the hydraulic oil in the hydraulic circuit, which connects the spaces below or above the pistons of single-acting or double-acting hydraulic cylinders, it is very easy to achieve the same magnitudes of pulling forces in the ropes.

In this article, double-acting hydraulic cylinders are used to carry out experiments with the spaces above the pistons interconnected by a hydraulic pipe with only hydrostatic pressure. If single-acting hydraulic cylinders were used, then the experimental measurements carried out on the laboratory equipment would show absolutely identical results, provided that the hydraulic oil pressure supplied to the space above the hydraulic cylinder pistons would be half the magnitude of the pressure delivered to the space above the hydraulic cylinder pistons. The internal cross-section of the hydraulic cylinder (space above the piston) is larger; see S_c_ [m^2^] and S_hc_ [m^2^] (2). The magnitude of the pulling force in the rope F_Rc_ = F_R_·k_1_ [N] (3), where k_1_ = S_c_/S_hc_.

One unique contribution of this article—and a new finding—is the set of tables listing the values of the measured pulling forces in ropes when the spaces under the pistons of the hydraulic cylinders were connected to each other. The new findings can be observed in the attached graphs of the measured pulling forces in the ropes in the individual phases of the experiments, which made it possible to specify all the conditions in order to be able to declare that the initially different magnitudes of the pulling forces in the ropes become the same if the hydraulic device specified in this article is used.

## Figures and Tables

**Table 3:** The pulling force in the rope and the pressure of the hydraulic oil in the space under the hydraulic cylinder piston and in the supply pipe.

n	F_p_	F_R1_	p_p_	p_p1_	F_R1p_	p_p1p_
	[N]		[MPa]		[N]	[MPa]
1	653.0 ^1^	1158.7 ^2^	1.33	2.36	1036.0 ^3^	2.11
2	285.9	618.5	0.58	1.26	593.7	1.21
3	406.5	938.2	0.83	1.91	912.4	1.86

^1^ see Figure 13a; ^2^ see Figure 13b; ^3^ see Figure 13c.

**Table 5:** The pulling force in the rope and the pressure of the hydraulic oil in the space under the hydraulic cylinder piston and in the supply pipe.

n	F_R1p1_	F_R2R1p1_	F_R1p1R2_	F_R3R2_	F_R3R2R1p_	F_R1pR2R3_	F_R2R1pR3_	F_1_	F_2_	F_3_
	[N]									
1	593.0 ^1^	947.6 ^2^	604.7 ^2^	1867.4 ^2^	1170.7 ^3^	749.5 ^3^	961.5 ^3^	184.1 ^4^	200.1 ^4^	178.0 ^4^
2	1034.5	955.6	1170.2	1656.7	1005.4	1170.2	1358.9	534.5	556.5	503.4
3	1104.0 ^5^	1170.2 ^6^	940.2 ^6^	1766.0 ^6^	1766.0 ^7^	955.9 ^7^	1170.2 ^7^	169.3 ^8^	183.4 ^8^	158.5 ^8^

^1^ see Figure 15a; ^2^ see Figure 15b; ^3^ see Figure 15c; ^4^ see Figure 15d; ^5^ see Figure 16a; ^6^ see Figure 16b; ^7^ see Figure 16c; ^8^ see Figure 16d.

## Data Availability

The measured data of force values F_Ri_ [N], listed from Table 1, Table 2, Table 3, Table 4 and Table 5 and processed using DEWESoft^®^ X2 SP5 software, can be sent in the case of interest, by prior written agreement, in *.XLSX (Microsoft Excel Office 365) format.
